# External validation of life expectancy prognostic models in patients evaluated for palliative radiotherapy at the end‐of‐life

**DOI:** 10.1002/cam4.3257

**Published:** 2020-06-26

**Authors:** Adrianna E. Mojica‐Márquez, Joshua L. Rodríguez‐López, Ankur K. Patel, Diane C. Ling, Malolan S. Rajagopalan, Sushil Beriwal

**Affiliations:** ^1^ Universidad Central del Caribe School of Medicine Bayamón PR USA; ^2^ Department of Radiation Oncology UPMC Hillman Cancer Center University of Pittsburgh School of Medicine Pittsburgh PA USA; ^3^ Department of Radiation Oncology Mount Carmel Health System Columbus OH USA

**Keywords:** prognosis, radiation therapy, radiotherapy, survival

## Abstract

**Background:**

The TEACHH and Chow models were developed to predict life expectancy (LE) in patients evaluated for palliative radiotherapy (PRT). We sought to validate the TEACHH and Chow models in patients who died within 90 days of PRT consultation.

**Methods:**

A retrospective review was conducted on patients evaluated for PRT from 2017 to 2019 who died within 90 days of consultation. Data were collected for the TEACHH and Chow models; one point was assigned for each adverse factor. TEACHH model included: primary site of disease, ECOG performance status, age, prior palliative chemotherapy courses, hospitalization within the last 3 months, and presence of hepatic metastases; patients with 0‐1, 2‐4, and 5‐6 adverse factors were categorized into groups (A, B, and C). The Chow model included non‐breast primary, site of metastases other than bone only, and KPS; patients with 0‐1, 2, or 3 adverse factors were categorized into groups (I, II, and III).

**Results:**

A total of 505 patients with a median overall survival of 2.1 months (IQR: 0.7‐2.6) were identified. Based on the TEACHH model, 10 (2.0%), 387 (76.6%), and 108 (21.4%) patients were predicted to live >1 year, >3 months to ≤1 year, and ≤3 months, respectively. Utilizing the Chow model, 108 (21.4%), 250 (49.5%), and 147 (29.1%) patients were expected to live 15.0, 6.5, and 2.3 months, respectively.

**Conclusion:**

Neither the TEACHH nor Chow model correctly predict prognosis in a patient population with a survival <3 months. A better predictive tool is required to identify patients with short LE.

## Graphical abstract

In patients who died within 90 days of consultation for palliative radiotherapy, the TEACHH and Chow models inadequately identified patients at risk of short‐term mortality. Better prognostic models are required to minimize use of unnecessary oncologic treatments among patients seen at the end‐of‐life.

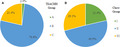

## 1 INTRODUCTION

The ability to accurately predict prognosis in patients with advanced cancer is a challenging task, which has important implications on treatment recommendations. It is well‐recognized that physicians tend to overestimate survival.^1^ A recent prospective study demonstrated that radiation oncologists have a prognostication accuracy of 40%.^2^ Inaccurate prognostication may lead to inappropriate treatment recommendations, associated complications, and undue burden on patients who may spend a significant proportion of their remaining life on undergoing oncologic treatment.^3^, ^4^ However, palliative treatment at the end‐of‐life may ease troublesome symptoms and is worthwhile in appropriately selected patients to improve quality of life.

Palliative radiotherapy (PRT) is important for symptom control in patients with advanced cancer,^5^ and patients undergoing PRT comprise approximately 20%‐50% of all patients undergoing treatment in radiation oncology departments.^6^ Despite national guidelines supporting the use of short‐course (SCRT) or single‐fraction radiotherapy (SFRT) in the palliative setting, long‐course PRT is commonly utilized at the end‐of‐life.^7^ An analysis of the Surveillance, Epidemiology, and End Results (SEER) data demonstrated that fewer than 10% of patients who received PRT during their last 30 days of life were treated with SFRT, whereas 17.8% received more than 10 days of PRT.^8^ Recommendations for protracted PRT courses in patients at the end‐of‐life could be related to inaccurate life expectancy (LE) predictions, as the factor most frequently influencing a dose‐fractionation prescription is patient prognosis.^9^ However, other factors have been identified (e.g. younger age, female sex, primary cancer diagnosis, no brain metastases, and private insurance).^10^ For this reason, LE tools have been proposed to estimate survival and help physicians in their clinical decision‐making.

Chow et al developed and validated a number of risk factors (NRF) model to predict patient prognosis in a cohort of patients who received PRT at two centers in Toronto, Ontario, Canada.^11^, ^12^, ^13^ Patients were assigned one point for each of three adverse features: nonbreast primary, site of metastases other than bone only, and Karnofsky performance status (KPS) ≤60. The model stratifies patients by number of adverse features (0‐1, 2, or 3) into three prognostic groups (Groups I, II, and III, respectively) with distinct median survivals: 15.0 months (95% Confidence Interval [CI]: 9.3‐17.5) vs 6.5 months (95% CI: 5.0‐7.8) vs 2.3 months (95% CI: 1.5‐2.8), respectively. The TEACHH model proposed by Krishnan et al was designed to identify patients with short (<3 months) and long LE (>1 year).^14^ The model assigns one point for each adverse prognostic factor including lung or other nonbreast and nonprostate primary, Eastern Cooperative Oncology Group (ECOG) performance status 2+, age >60, >2 courses of prior palliative chemotherapy, a prior hospitalization within the last 3 months, and presence of hepatic metastases. Patients are classified by number of adverse factors (0‐1, 2‐4, or 5‐6) into three prognostic groups (Groups A, B, and C, respectively) with distinct median survivals: 19.9 months (95% CI: 13.9‐31.1) vs 5.0 months (95% CI: 4.3‐5.6) vs 1.7 months (95% CI 1.2‐2.1), respectively.

Although the Chow and TEACHH models assist physicians with predicting LE in patients with advanced cancer, both models were developed using patient cohorts seen at academic centers, with relatively long predicted LE, and included few patients with predicted LE less than 3 months (33.0% and 5.7%, respectively). The median survivals in the Chow and TEACHH patient cohorts were 4.9 and 5.6 months, respectively. Thus, these models may not accurately predict prognosis in patients with a short LE less than 3 months. It is important to accurately identify these patients, as they are most at risk of receiving unnecessarily protracted PRT courses and may benefit from hospice or SFRT/SCRT to minimize logistical burdens and toxicity associated with radiotherapy. As such, we sought to validate the TEACHH and Chow models in patients who were evaluated for PRT and died within 90 days of their initial encounter. We hypothesize that the models will adequately stratify this patient population into the respective groups with limited survival.

## 2 METHODS AND MATERIALS

An IRB‐approved retrospective cohort study was performed on consecutive patients who were evaluated for PRT within a large integrated cancer network between January 2017 and August 2019 and died within 90 days of their encounter. Patients were excluded if they were treated with definitive or salvage intent. Electronic charts were reviewed for information pertaining to demographics, clinical, and treatment‐related characteristics. Data were obtained from integrated sites through the Aria database (Varian Medical Systems).

Patient records were reviewed for components of the TEACHH model including type of cancer (breast or prostate vs lung or other), ECOG performance status (0‐1 vs 2‐4), age (≤60 or >60 years), number of prior palliative chemotherapy courses (0‐2 vs >2), prior hospitalizations within the previous 3 months (0 vs ≥1), and hepatic metastases (present or absent), assigning one point for each adverse prognostic factor (Table 1). The point system has a minimum score of zero and a maximum of six points. Patients with 0‐1 risk factors were categorized into Group A, 2‐4 risk factors into Group B, and 5‐6 risk factors into Group C.

**TABLE 1:** TEACHH score calculation among patients dying within 90 d

Adverse factor	Points Assigned	No. % (n = 505)
Type of primary tumor		
Breast or Prostate	0	87 (17.2)
Lung or other	1	418 (82.8)
ECOG performance status		
0‐1	0	264 (52.3)
2‐4	1	241 (47.7)
Age (y)		
≤60	0	129 (25.5)
>60	1	376 (74.5)
Prior lines of palliative chemotherapy		
≤2	0	216 (42.8)
>2	1	289 (57.2)
Hepatic metastases		
Absent	0	316 (62.6)
Present	1	189 (37.4)
Hospitalizations in prior 3 mo		
No	0	179 (35.4)
Yes	1	326 (64.6)
Group (median life expectancy)		
A (19.9 mo)	0‐1	—
B (5.0 mo)	2‐4	—
C (1.7 mo)	5‐6	—

Abbreviation: ECOG, Eastern Cooperative Oncology Group.

The components of the Chow model were also collected, which include cancer type (breast vs non‐breast), KPS (>60 vs ≤60), and metastasis location (bone only vs other). The number of risk factors was counted for each patient: non‐breast primary cancer, site of metastasis other than bone, and KPS ≤ 60 (Table 2). Patients with 0‐1 risk factors were categorized into Group I, 2 risk factors into Group II, and 3 risk factors into Group III.

**TABLE 2:** Chow score calculation among patients dying within 90 d

Adverse factor	Points assigned	No. % (n = 505)
Primary cancer site		
Breast	0	55 (10.9)
Other	1	450 (89.1)
Site of metastases		
Bone only	0	150 (29.7)
Other	1	355 (70.3)
KPS		
>60	0	264 (52.3)
≤60	1	241 (47.7)
Group (median life expectancy)		
I (15.0 mo)	0‐1	—
II (6.5 mo)	2	—
III (2.3 mo)	3	—

Abbreviation: KPS, Karnofsky Performance Status.

### 2.1 Statistical methods

Data analysis was performed with IBM SPSS Statistics for Windows, version 25 (IBM Corp., Armonk, NY). Demographics, clinical, treatment‐related characteristics, and model's accuracy are described with descriptive statistics. Time end point was defined as time to event from PRT appointment date. Univariate analysis (UVA) using Cox regression was performed to identify predictors of 30‐day mortality after PRT consultation as these patients were seen near the end‐of‐life. Variables with a significance level of *P* < .10 on UVA were enter into a multivariate model using the forward conditional method. Statistical significance was established at a *P* value of ≤.05.

## 3 RESULTS

A total of 505 patients were evaluated for PRT and died within 90 days. The median overall survival was 2.1 months (interquartile range [IQR], 0.7‐2.6). The median age was 67 years (IQR: 60‐75). Patient, tumor, and treatment‐related characteristics are shown in Table 3. Most patients were Caucasian (90.5%), male (52.7%), had primary lung cancer (51.7%), and were treated for bone metastases (50.1%). The majority of patients were evaluated at community practices (61.2%). [Correction added on 22 July 2020, after first online publication: in the preceding sentence, 73.5% was corrected to 61.2%.]. PRT was recommended to 481 patients (95.4%), of which ≥10 fractions were recommended to 315 (65.5%) patients. Of 429 patients who began a course of PRT, a total of 95 (22.1%) patients did not complete PRT.

**TABLE 3:** Baseline characteristics of entire cohort

Baseline characteristics	All patients (N = 505) n (%)
Diagnosis	
Brain Metastasis	164 (32.5)
Bone Metastasis	253 (50.1)
Other	88 (17.4)
Treatment recommendation	
None	24 (4.8)
SFRT (1 Fx)	41 (8.1)
SCRT (2‐9 Fxs)	82 (16.2)
LCRT (≥10 Fxs)	304 (60.2)
SAbR	54 (10.7)
Age (y)	
≤67	259 (51.3)
>67	246 (48.7)
Race	
Caucasian	457 (90.5)
African‐American	40 (7.9)
Other/Unknown	8 (1.6)
Ethnicity	
Non‐Hispanic or Latino	492 (97.4)
Other/Unknown	13 (2.6)
Sex	
Male	266 (52.7)
Female	239 (47.3)
Practice setting	
Community	309 (61.2)
Academic	196 (38.8)
Primary site	
Lung	261 (51.7)
Breast	55 (10.9)
Prostate	32 (6.3)
Other	157 (31.1)
Performance status	
90‐100, ECOG 0	42 (8.3)
70‐80, ECOG 1	192 (38.0)
50‐60, ECOG 2	171 (33.9)
30‐40, ECOG 3	48 (9.5)
10‐20, ECOG 4	7 (1.4)
Unrecorded	45 (8.9)
Inpatient consult	
No	371 (73.5)
Yes	134 (26.5)
Time of death (d)	
Within 0‐30	193 (38.2)
Within 31‐60	28 (5.5)
Within 61‐90	284 (56.2)

Abbreviations: ECOG, Eastern Cooperative Oncology Group; LCRT, Long‐course Radiotherapy; SAbR, Stereotactic Ablative Radiotherapy; SCRT, Short‐course Radiotherapy; SFRT, Single‐fraction Radiotherapy. [Correction added on 22 July 2020, after first online publication: The data under subheading 'Practice setting' and 'Performance status' have been corrected, and the subheading 'Inpatient consult' has been added in this version]

### 3.1 Validation of the TEACHH model

**FIGURE 1:**
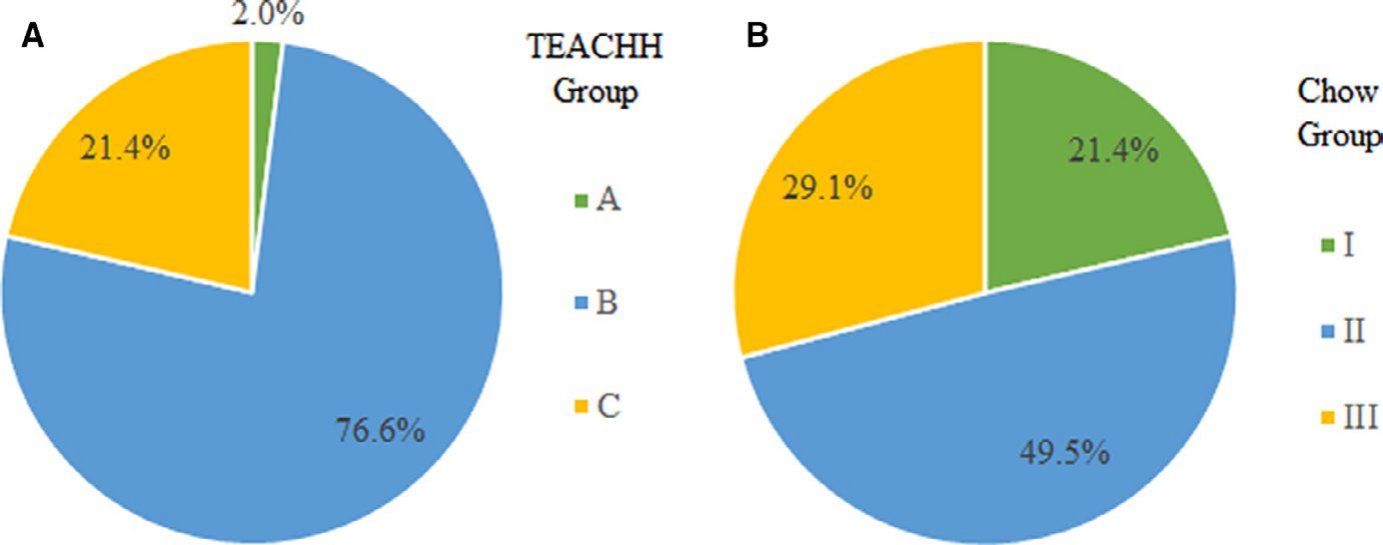
Distribution of patients by (A) TEACHH and (B) Chow models survival groups in a patient cohort surviving <3 mo

The majority of patients had a primary originating from lung or other (82.8%) instead of breast or prostate. Most patients were >60 years old (74.5%), had an ECOG performance status of 0‐1 (52.3%), received > 2 palliative chemotherapy regimens (57.2%), and had a prior hospitalization within 3 months of radiation oncology consultation (64.6%). Utilizing the TEACHH model calculation, 10 (2.0%), 387 (76.6%), and 108 (21.4%) patients were categorized into Group A, Group B, and Group C, respectively (Figure 1A). Herein, survival was correctly predicted for 21.4% of patients. Of patients categorized into Group C with a predicted median survival of 1.7 months per the TEACHH model, 56.5% died within 30 days. Table 4 outlines patient characteristics among TEACHH model groups.

**TABLE 4:** Patient characteristics according to TEACHH model prognostic group

Characteristic	Group A (n = 10)	Group B (n = 387)	Group C (n = 108)
Type of primary tumor			
Breast or Prostate	4 (40.0%)	73 (18.9%)	10 (9.3%)
Lung or Other	6 (60.0%)	314 (81.1%)	98 (90.7%)
ECOG performance status			
0‐1	10 (100.0%)	239 (61.8%)	15 (13.9%)
2‐4	0 (0.0%)	148 (38.2%)	93 (86.1%)
Age (y)			
≤60	8 (80.0%)	115 (29.7%)	6 (5.6%)
>60	2 (20.0%)	272 (70.3%)	102 (94.4%)
Prior lines of palliative chemotherapy			
≤2	9 (90.0%)	182 (47.0%)	25 (23.1%)
>2	1 (10.0%)	205 (53.0%)	83 (76.9%)
Hepatic metastases			
Absent	10 (100.0%)	277 (71.6%)	29 (26.9%)
Present	0 (0.0%)	110 (28.4%)	79 (73.1%)
Hospitalization in prior 3 mo			
No	10 (100.0%)	161 (41.6%)	8 (7.4%)
Yes	0 (0.0%)	226 (58.4%)	100 (92.6%)
Time of death (d)			
Within 0‐30	2 (20.0%)	130 (33.6%)	61 (56.5%)
Within 31‐60	5 (50.0%)	21 (5.4%)	2 (1.9%)
Within 61‐90	3 (30.0%)	236 (61.0%)	45 (41.7%)

Abbreviation: ECOG, Eastern Cooperative Oncology Group.

### 3.2 Validation of the Chow model

Most patients had a KPS > 60 (52.3%), a non‐breast primary (89.1%), and a non‐bone metastasis (70.3%). Utilizing the Chow model calculation, 108 (21.4%), 250 (49.5%), and 147 (29.1%) of patients were categorized into Group I, Group II, and Group III, respectively (Figure 1B). Of patients categorized into Group III with a predicted median survival of 2.3 months per the Chow model, 49.7% died within 30 days, see Table 5. The Chow model correctly predicted survival among 29.1% of patients.

**TABLE 5:** Patient characteristics according to Chow model prognostic group

Characteristic	Group I (n = 108)	Group II (n = 250)	Group III (n = 147)
Primary cancer site			
Breast	36 (33.3%)	19 (7.6%)	0 (0.0%)
Other	72 (66.7%)	231 (92.4%)	147 (100.0%)
Site of metastases			
Bone only	84 (77.8%)	66 (26.4%)	0 (0.0%)
Other	24 (22.2%)	184 (73.6%)	147 (100.0%)
KPS			
>60	99 (91.7%)	165 (66.0%)	0 (0.0%)
≤60	9 (8.3%)	85 (34.0%)	147 (100.0%)
Time of death (d)			
Within 0‐30	30 (27.8%)	90 (36.0%)	73 (49.7%)
Within 31‐60	5 (4.6%)	11 (4.4%)	12 (8.2%)
Within 61‐90	73 (67.6%)	149 (59.6%)	62 (42.2%)

Abbreviation: KPS, Karnofsky Performance Status. [Correction added on 22 July 2020, after first online publication: The data under the subheading 'KPS' have been corrected in this version]

### 3.3 Predictors of 30‐day mortality

UVA and multivariate analysis (MVA) were conducted to identify predictors of 30‐day mortality. On UVA, inpatient consult and hepatic metastases were negative predictors for 30‐day mortality (Table S1), and these remained significant on MVA (Table S2).

## 4 DISCUSSION

Our analysis has several important findings. In a patient cohort with a median survival of approximately 2 months, neither the Chow nor TEACHH models provided accurate prognostication. Nearly 80% of patients were classified into prognostic groups with predicted survivals of at least 5 months per the TEACHH model, and nearly a quarter of patients were predicted to survive 15 months per the Chow model. Of patients who began a course of PRT, nearly a quarter did not complete their prescribed regimen.

Several predictive models exist to assist physicians in LE prediction. Currently, there is no standardized model to predict LE in patients with advanced cancer being evaluated for PRT. Broader use of predictive models may allow for personalized treatment consistent with patients’ needs, values, and preferences.^12^, ^13^, ^14^, ^15^, ^16^ We decided to use the TEACHH and Chow models because both were developed in patients receiving PRT and incorporate cancer‐specific clinical variables easily accessible to the radiation oncologist at the time of consultation. In addition, the models are not limited to a specific disease site and have been validated externally in independent patient populations.^12^, ^13^, ^17^

As expected, our cohort represented patients with more advanced disease than the cohorts used to develop the TEACHH and Chow models. Patients with long LE were included in the development of both models. As a result, the median survivals in the Chow and TEACHH patient cohorts (4.9 and 5.6 months, respectively) were higher than our median survival of 2.1 months. Compared to the TEACHH training model cohort, our patients were more likely to have received > 2 palliative chemotherapy regimens (57.2% vs 17.1%), be older than 60 (74.5% vs 55.5%), have hepatic metastases (35.4% vs 23.6%), and have been hospitalized within the last 3 months (64.6% vs 55.3%). Compared to the Chow model cohort, our patients were less likely to have a breast primary (10.9% vs 20.0%). Another consideration is that 61.2% of our patient cohort were seen at community practices, whereas the cohorts used to develop the models were from academic practices. [Correction added on 22 July 2020, after first online publication: in the preceding sentence, 73.5% was corrected to 61.2%.] It is unclear whether palliative patients seen in the community are different from those seen at an academic center.

The TEACHH score correctly predicted LE in only 108 (21.4%) patients within our cohort. A possible explanation is that patients with short survival (Group C) compromised only 6% of the total cohort used to develop the TEACHH model; therefore, the model may not be accurate for patients near the end‐of‐life. On the contrary, LE was correctly predicted in 147 (29.1%) patients utilizing the Chow model, possibly because the Chow training model cohort had a larger proportion of patients (33%) stratified into Group III. Similar findings were reported by Wu et al, in patients who died within 30 days of completing radiotherapy.^18^ Only 24% and 55% of their patients were grouped into TEACHH Group C and Chow Group III. Unfortunately, as both models were developed using a patient cohort with relatively longer LE, they do not appear to be sufficiently accurate in identifying patients with short‐term survival less than 3 months.

Other comprehensive models that integrate clinical laboratory values may more accurately predict life expectancy in patients near the end‐of‐life. One such example is the Good Samaritan NEAT model, which has not been externally validated.^19^ The NEAT model evaluates number of active tumors (“N”), ECOG performance status (“E”), albumin (“A”), and primary tumor site (“T”). Four groups were identified, with median survivals ranging from 1.2, 4.1, 14.5, and >31.4 months.^19^ Also, the Prognosis in Palliative care Study (PiPS) predictor model assesses prognosis in patients with advanced cancer, and it is composed of four prognostic models reliable to identify patients with expected prognoses of “days,” “weeks,” or “months,” and could be integrated with laboratory values.^20^ However, the model was developed among patients not receiving active treatment for their cancer. Finally, the Imminent Mortality Predictor in Advanced Cancer (IMPAC) predicts short‐term mortality among hospitalized patients. The model had 60% positive predictive value for mortality within 90 days.^21^ Additionally, machine learning algorithms could be incorporated into clinical practice to facilitate prognostication and clinical decision‐making. Machine learning algorithms were recently shown to predict 180‐day mortality from the index encounter with high accuracy.^22^ LE predictive models may be a helpful tool for all oncologists to facilitate timely conversations with patients regarding goals of treatment, especially in patients with short survival as patients can have inaccurate perceptions of their prognoses and are prone to receive aggressive treatments.^23^, ^24^

Our study is unique given that we only included patients who were evaluated at the end‐of‐life. Limitations of this study include its retrospective nature, which is subject to reporting bias. A major limitation of our study was the need to rely on clinical information from the history and physical examination to estimate performance status in patients for whom a performance status was not explicitly recorded at time of initial encounter (8.9%). In addition, performance status scales are subject to bias and ultimately have a significant impact on risk factor scoring and LE group assignment. Finally, our study incorporates data from a large, integrated cancer network, composed of academic and community practices and served by multiple providers, which results in a potential for interobserver variability in determining performance status. Both the TEACHH and Chow models should be validated in a prospective fashion using a cohort of patients near the end‐of‐life; however, this may be difficult due to the poor prognosis in this vulnerable population.

## 5 CONCLUSION

The TEACHH and Chow models inadequately identified patients at risk of short‐term mortality. A more comprehensive predictive model that identifies patients near the end‐of‐life is required to avoid unnecessary treatment and improve quality of patient care.

## DATA SHARING STATEMENT

All data generated and analyzed during this study are included in this manuscript.

## CONFLICT OF INTEREST

Sushil Beriwal, MD, MBA – Director of Elsevier ClinicalPath formerly Via Oncology; Advisor Varian.

## AUTHOR CONTRIBUTIONS

SB participated in project conceptualization, supervision, and writing‐review editing. SB, AMM, and JRL participated in data curation, methodology, and formal analysis. AMM and JRL participated in writing of original draft. AP participated in methodology, formal analysis, and writing‐review and editing. DL and MR participated in methodology and writing‐review editing.

## Supporting information

Table S1‐S2
