# Supplementary material for: External validation of life expectancy prognostic models in patients evaluated for palliative radiotherapy at the end‐of‐life
Source: Cancer Med. 2020 Jun 26;9(16):5781–7. doi: 10.1002/cam4.3257 (PMC7433812; doi:10.1002/cam4.3257)
Supplement: Supplementary file 1 — Table S1‐S2 [file CAM4-9-5781-s001.docx]

**Supplemental Table 1:** Univariate Analysis of 30-Day Mortality

|  | **No**  **(n=312)** | **Yes**  **(n=193)** | **HR** | **95% CI** | ***P*** |
| --- | --- | --- | --- | --- | --- |
| **Inpatient Consult**  No  Yes  **Hospitalization prior 3 months**  No  Yes  **Sex**  Male  Female  **Race**  Caucasian  African-American  Other/Unknown  **Age (years)**  ≤ 67  > 67  **Diagnosis**  Brain metastases  Bone metastases  Other  **Lines of Palliative Chemo**  ≤2  >2  **Type of Primary Tumor**  Breast or Prostate  Lung or other  **Hepatic Metastases**  Absent  Present  **ECOG Performance Status**  0-1  2-4  **TEACHH Score Group**  A  B  C  **Chow Score Group**  I  II  III | 218 (58.8%)  94 (70.1%)    99 (55.3%)  213 (65.3%)    160 (60.2%)  152 (63.6%)    279 (61.1%)  27 (67.5%)  6 (75.0%)    145 (56.0%)  167 (67.9%)    104 (63.4%)  159 (62.8%)  49 (55.7%)  133 (61.6%)  179 (61.9%)  49 (56.3%)  263 (62.9%)    169 (53.5%)  143 (75.7%)    148 (56.1%)  164 (68.0%)    5 (50.0%)  199 (51.4%)  108 (100.0)  64 (59.3%)  138 (55.2%)  110 (74.8%) | 153 (41.2%)  40 (29.9%)    80 (44.7%)  113 (34.7%)    106 (39.8%)  87 (36.4%)    178 (38.9%)  13 (32.5%)  2 (25.0%)    114 (44.0%)  79 (32.1%)    60 (36.6%)  94 (37.2%)  39 (44.3%)  83 (38.4%)  110 (38.1%)  38 (43.7%)  155 (37.1%)    147 (46.5%)  46 (24.3%)    116 (43.9%)  77 (32.0%)    189 (38.4%)  4 (30.8%)  0 (0.0%)  44 (40.7%)  112 (44.8%)  37 (25.2%) | 1  0.61    1  0.76    1  0.90    1  0.72  0.42    1  0.89    1  1.04  1.29  1  0.81  1  0.88    1  0.57    1  1.13    1  0.74  -  1  1.25  0.96 | Reference  0.43-0.86    Reference  0.57-1.02    Reference  0.68-1.20  Reference  0.41-1.26  0.10-1.70    Reference  0.67-1.19    Reference  0.75-1.43  0.86-1.94  Reference  0.61-1.07  Reference  0.62-1.26    Reference  0.41-0.79    Reference  0.85-1.52    Reference  0.30-1.79  -    Reference  0.88-1.77  0.62-1.49 | <0.01  0.07  0.48  0.25  0.42  0.41  0.14  0.50  <0.01  0.40  0.78  0.26 |
| Abbreviations: HR, Hazards Ratio; CI, Confidence Interval; Chemo, Chemotherapy; ECOG, Eastern Cooperative Oncology Group | | | | | |

**Supplemental Table 2:** Multivariate Analysis of 30-Day Mortality

| **Significant characters** | **HR (95% CI)** | ***P*** |
| --- | --- | --- |
| **Hepatic Metastases**  Absent  Present  **Inpatient Consult**  No  Yes | Reference  0.58 (0.42-0.81)  Reference  0.63 (0.44-0.89) | <0.01  <0.01 |
| Abbreviations: HR, Hazards Ratio; CI, Confidence Interval | | |
